# Nonalcoholic Fatty Liver Disease and Cardiovascular Diseases: The Heart of the Matter

**DOI:** 10.1155/2021/6696857

**Published:** 2021-01-12

**Authors:** Stefan Chiriac, Carol Stanciu, Irina Girleanu, Camelia Cojocariu, Catalin Sfarti, Ana-Maria Singeap, Tudor Cuciureanu, Laura Huiban, Cristina Maria Muzica, Sebastian Zenovia, Robert Nastasa, Anca Trifan

**Affiliations:** ^1^Department of Gastroenterology, “Grigore T. Popa” University of Medicine and Pharmacy, Iasi 700115, Romania; ^2^Institute of Gastroenterology and Hepatology, “St. Spiridon” Emergency Hospital, Iasi 700111, Romania

## Abstract

Nonalcoholic fatty liver disease (NAFLD) has emerged as the most frequent cause of liver disease worldwide, comprising a plethora of conditions, ranging from steatosis to end-stage liver disease. Cardiovascular disease (CVD) has been associated with NAFLD and CVD-related events represent the main cause of death in patients with NAFLD, surpassing liver-related mortality. This association is not surprising as NAFLD has been considered a part of the metabolic syndrome and has been related to numerous CVD risk factors, namely, insulin resistance, abdominal obesity, dyslipidemia, hyperuricemia, chronic kidney disease, and type 2 diabetes. Moreover, both NAFLD and CVD present similar pathophysiological mechanisms, such as increased visceral adiposity, altered lipid metabolism, increased oxidative stress, and systemic inflammation that could explain their association. Whether NAFLD increases the risk for CVD or these diagnostic entities represent distinct manifestations of the metabolic syndrome has not yet been clarified. This review focuses on the relation between NAFLD and the spectrum of CVD, considering the pathophysiological mechanisms, risk factors, current evidence, and future directions.

## 1. Introduction

In the last decades, the prevalence of nonalcoholic liver disease (NAFLD) has been rapidly increasing [1]. NAFLD affects from 25 to 45% of the general adult population and up to 70% of type 2 diabetic patients in Europe and North America [2]. Nonalcoholic steatohepatitis (NASH) is a subtype of NAFLD characterized by progressive liver disease that can lead to liver cirrhosis and hepatocellular carcinoma. Most of the patients with NAFLD develop mild disease, although 20–30% of them progress to NASH [2]. Approximately 20% of the patients with NASH and progressive fibrosis will develop liver cirrhosis with an increased risk of hepatocellular carcinoma [3–5].

NAFLD is associated with multiple extrahepatic diseases ranging from mild to severe organ-specific-related complications. Patients with NAFLD usually associate features of metabolic syndrome (MetS) which overlaps with the cardiovascular risk factors. All these factors are involved in the development of cardiovascular events (CVEs), which are the most common causes of death among these patients [6]. Several prospective and retrospective studies confirmed the association between NAFLD and cardiovascular diseases (CVD), with a negative impact on patients' outcome [7, 8].

All these studies have investigated the association between NAFLD and CVD, and efforts have been made to establish a direct relationship between these two complex conditions. However, as both are multifactorial diseases sharing common risk factors, a direct causality relation between NAFLD and the development of CVD has not yet been firmly established [9]. Of note, a previous meta-analysis demonstrated that the risk of developing a CVEe is 64% higher in patients with vs. without NAFLD [10]. The method of NAFLD diagnosis (ultrasound or computed tomography) was the main limitation of the studies included in this meta-analysis. The lack of a histologically proven diagnosis of NAFLD in the cross-sectional studies regarding cardiovascular involvement maintains the controversy on whether NAFLD is an active contributor or an innocent bystander in CVD development.

The complex physiopathology of both diseases with common risk factors and simultaneous involvement of different pathways makes it difficult to draw a clear conclusion regarding the direct relationship between NAFLD and CVD. Whether NAFLD confers any additional CVD risk, or whether an increase in CVD risk in NAFLD is due to associated CVD risk factors, is still uncertain. Confirming if NAFLD contributes as an independent CVD risk factor is important, as it is plausible that treatment of liver disease may decrease the CVD risk.

The aim of this review is to provide an update on the clinical evidence that supports the association between NAFLD and CVD, the impact on disease outcome, the main pathophysiological mechanism, and the most common cardiovascular comorbidities.

## 2. Pathophysiology of Cardiovascular Involvement in NAFLD

**Figure 1:**
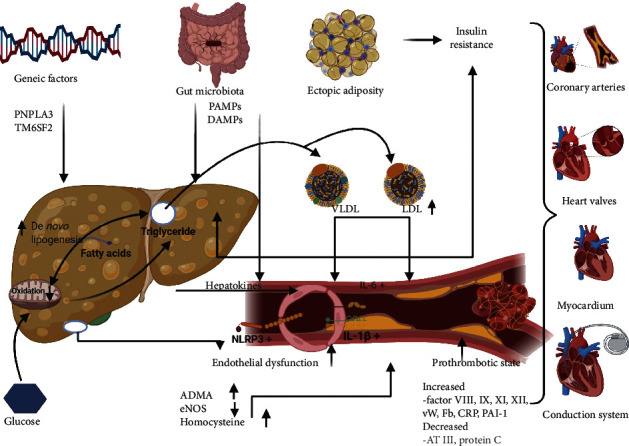
Pathophysiology of cardiovascular involvement in NAFLD.

The pathophysiology behind the association of NAFLD with CVD is still incompletely understood and may involve other pathways besidesinsulin resistance (IR), such as oxidative stress, inflammation, and gut microbiota (Figure 1).

*Abnormal glucose metabolism and hepatic IR* are the major hallmarks of NAFLD, and they are the main elements in NAFLD and CVD pathogenesis [11–13]. The glucose metabolism disorders in NAFLD are secondary to the underlying systemic inflammation, visceral adiposity, and ectopic fatty tissue [14, 15]. The IR is associated with hyperinsulinemia that determines increase in hepatic glucose production and chronic hyperglycemia. Persistent hyperglycemia and IR promote oxidative stress, activation of inflammation, and dysregulation of lipoprotein metabolism [14, 16, 17]. IR promotes oxidative stress and activation of inflammatory signaling pathways, vascular inflammation, and dysregulation of lipids metabolism contributing to ectopic fat accumulation [14, 18, 19]. Pancreatic ectopic adipose tissue is also associated with IR and beta cell dysfunction, hyperinsulinemia, and secondary increase of free fatty acid level. Hyperinsulinemia and the decrease of hepatic insulin clearance secondary to NAFLD are associated with increased hepatic gluconeogenesis, hyperglycemia, and insulin overproduction, a pathological self-reinforcing cycle. Insulin, as a catabolic hormone, increases the production of various lipogenic enzymes by activating transcription factors as carbohydrate-responsive element binding protein (ChREBP) or sterol regulatory element-binding proteins 1c (SREBP-1c) [20]. The consequence is further accumulation of hepatic fat, overproduction of VLDL particles, and increasing the vascular atherogenetic process.

*Atherogenic dyslipidemia* associated with NAFLD is the consequence of increased de novo hepatic lipogenesis along with an elevated rate of lipid uptake, both mechanisms determining the overproduction and secretion of large triglyceride-enriched VLDL particles, including apolipoprotein C3 (ApoC3) and apolipoprotein B (ApoB). The atherogenic dyslipidemia is characterized by high serum triglycerides, low high-density lipoprotein (HDL) cholesterol, the predominance of small dense low-density lipoprotein (LDL) particles, and increased intermediate-density lipoprotein (IDL) [14, 21]. The atherogenic lipoproteins penetrate the vascular wall and activate the toll-like receptors (TLRs). These receptors sense the endogen damage signals and activate an immune response [22]. Activation of TLRs 2 and 4 receptors has a primary impact on activation of NOD-like receptor family, pyrin domain-containing protein 3 (NLRP3) inflammasome [14, 23]. NLRP3 inflammasome regulates the activity of enzyme caspase-1, known as interleukin (IL)-1*β* converting enzyme [24]. This complex mechanism leads to the activation of proinflammatory cytokines as IL-1*β*, IL-6, and C-reactive protein (CRP), all of them being involved in vascular inflammation and promoting atherosclerotic cardiovascular disease [24]. NAFLD patients have also an increased level of palmitic acid, incorporated in VLDL, and this saturated fatty acid also induces vascular inflammation by activating TLRs 2 and 4 [14, 25].

*Endothelial dysfunction* is one of the most important pathophysiological links between NAFLD and cardiovascular diseases. The oxidative stress and lipoprotein-mediated vascular inflammation are related to endothelial dysfunction that is characterized by decreased bioavailability of the nitric oxide (NO) [14, 23, 26, 27]. There are two main factors contributing to endothelial dysfunction in NAFLD patients: low NO availability and hyperhomocysteinemia. It was demonstrated that patients with NAFLD have a low level of asymmetric dimethyl arginine (ADMA) determined by decreased liver breakdown of this molecule. ADMA is an endogenous antagonist of nitric oxide synthase (NOs) and its elevation is associated with decreased NO. Hyperhomocysteinemia induces oxidative stress by reduced glutathione stores in direct relation with low levels of NO. All these endothelial abnormalities increase platelet activation and vascular resistance [28–30].

In patients with NAFLD, an *imbalanced coagulation* cascade was demonstrated, and these subjects are being prone to a hypercoagulable state due to high levels of coagulation factors FVIII, FIX, FXI, FXII, fibrinogen, von Willebrand factor, and plasminogen activator inhibitor-1, along with low levels of anticoagulant factors as antithrombin III and protein C [12, 26, 30].

Recently, the hepatokines have been demonstrated to be potential mediators of cardiometabolic syndrome in NAFLD [31]. Of these, fetuin A was associated with CVD [32]. The experimental studies have demonstrated that fetuin A induces low-grade inflammation in concert with fatty acids [33].

Fat accumulation in the liver could be associated with ectopic fatty tissue, including myocardial fat and adipose tissue surrounding the heart, a central aspect of the relationship between NAFLD and CVD [34]. Under physiological conditions, this adipose tissue has anti-inflammatory and antifibrotic proprieties [35, 36]. In NAFLD, the systemic inflammatory syndrome is changing the epicardial adipose tissue phenotype, transforming these cells in activated adipose cells that secret proinflammatory cytokines, activate profibrotic pathways, and promote ventricular fibrosis and inflammation [35–37].

The previous studies demonstrated that NAFLD has also a *genetic predisposition*. The polymorphism in the patatin-like phospholipase domain-containing 3 (PNPLA3) and the transmembrane 6 superfamily member 2 (TM6SF2) genes are associated with NAFLD, NASH, fibrosis, and an increased risk of hepatocellular carcinoma [38]. PNPLA3 I148 M and TM6SF2 E167 K are variants that interfere with hepatic triglyceride metabolism [39, 40]. Both variants are predisposing the patients to NAFLD and are associated with increased disease severity [41]. Interestingly, carriers of genetic variants of PNPLA3 and TM6SF2 tend to have cardioprotective phenotype [42, 43], although the precise in vivo physiological role of PNPLA3 remains incompletely characterized.

Recently, *gut microbiota* was demonstrated as a contributing factor for atherosclerosis, T2DM, and NAFLD [44, 45]. The impaired gut mucosal barrier permits pathogen-associated molecular patterns (PAMPs) and damaged-associated molecular patterns (DAMPs) entering the systemic circulation, inducing a gut-related inflammatory response [46, 47]. NAFLD and advanced fibrosis are associated with an increased concentration of *Escherichia coli* bacteria, *Ruminococcus*, and *Blautia* and a decrease in *Firmicutes* strains [48–50]. This profound intestinal dysbiosis is independent of IR and obesity and is related to increased gut-derived metabolites as 3-(4-hydroxyphenyl)-lactate or phenylacetate. Also, gut-derived microbiota and its metabolites were recently demonstrated as potentially important players in cardiovascular disease pathophysiology [51].

All this evidence supports the important role of the liver in the pathophysiologic processes of CVD development, although an independent link between NAFLD and coronary arterial disease and atherosclerosis remains difficult to confirm.

## 3. Cardiovascular Risk Assessment in NAFLD

NAFLD shares many risk factors with CVD, most notably insulin resistance, obesity, and dyslipidemia. Also, NAFLD itself likely influences CVD development, by means of hypertriglyceridemia and induction of a hypercoagulable state [10, 30, 52].

Several studies demonstrated that all stages of NAFLD are associated with increased CVEs as acute coronary syndrome, stroke, or malignant arrhythmias. Moreover, compared with patients without NAFLD, those with fatty liver have an elevated risk of CVEs independent of the presence of MetS or T2DM [6, 53]. Even in normoponderal patients, ultrasound-defined NAFLD is correlated with a high incidence of CVEs, concluding that NAFLD acts independently of overweight and obesity [54].

Recent data also suggested that patients with NAFLD had a twofold increase in the risk of developing CVEs, and in those with liver fibrosis this risk was doubled [6].

As a systemic progressive disease, NAFLD increases the risk of CVD, although the most commonly used cardiovascular risk factor scoring system for cardiovascular risk management, such as the Framingham Risk Score or SCORE, may underestimate cardiovascular risk in this special patient category. No validated CVD risk score specific for NAFLD patients has yet been validated.

The most important clinical practice issue is that NAFLD diagnosis could be associated with an additional risk for CVD when concomitant atherosclerotic risk factors are already diagnosed, although before including NAFLD in new cardiovascular risk scores we should establish a consensus on how to quantify and qualify NAFLD severity. Until then, the use of classical risk factors is adequate to evaluate CVD risk in NAFLD patients, as Treeprasertsuk et al.demonstrated. In this study, the Framingham Risk Score had a good sensibility in identifying coronary heart disease risk in a cohort with more than 300 NAFLD patients followed by a mean of 11.5 years [55].

An important clinical question is if NAFLD indicates the need for an extensive cardiovascular risk assessment independently of the presence of classical risk factors. Many cross-sectional studies confirmed the independent associations between NAFLD and the presence of subclinical vascular disease or changes in heart morphology such as left ventricular hypertrophy or diastolic dysfunction. However, there is no prospective evaluation showing an additional role of these imaging tests in CVD risk evaluation. Therefore, there is not enough evidence to routinely recommend imaging tests for subclinical vascular or heart disease based on the presence of NAFLD. The association between NAFLD and CVD is further described in Table 1.

### 3.1. Impact of NAFLD on Cardiovascular Disease Outcome

Several meta-analyses reported conflicting results regarding cardiovascular mortality rate in patients with NAFLD. A meta-analysis including 16 studies demonstrated an increased risk of CVEs in NAFLD patients compared to those without NAFLD [10]. However, the CVD-related mortality was higher only in patients with NASH and high fibrosis scores or high histological fibrosis stage. A second meta-analysis found an increased liver-related mortality in patients with NAFLD, with no correlation with CVD-related mortality [72]. Moreover, a meta-analysis of 34 studies including more than 160,000 patients by Wu et al. was unable to confirm a correlation between the presence of NAFLD and increased cardiovascular mortality [73]. The major limitation of these meta-analyses lies in the heterogeneity of diagnosis criteria of NASH. However, in consideration of all studies, the meta-analysis of Wu et al. confirmed that NAFLD was associated with an increased risk for incident CVD (HR 1.37; 95% CI 1.10–1.72) and that NAFLD patients were more likely to develop coronary heart disease (HR 2.31; 95% CI 1.46–3.65) and hypertension (HR 1.16; 95% CI 1.06–1.27) [73]. In addition, it was demonstrated that the severity of NAFLD was a major determinant of increased risk of CVD [74]. A comprehensive meta-analysis performed by Younossi et al.that included 86 studies, with a sample size of 8,515,431 patients, reported a pooled CVD-related mortality rate in patients with NAFLD of 4.8 per 1000 person-years [75].

Current evidence shows that NAFLD is associated with an increased risk for CVD and CVEs. Patients with NASH and advanced fibrosis as well as NAFLD patients with concomitant T2DM can be identified as being part of a special risk group [76–78].

Ekstedt et al., in a study with a mean follow-up duration of 26.4 years, stated that patients with NAFLD presented higher mortality than patients from the general population (HR: 1.29; 95% CI 1.04–1.59) [43]. The authors identified CVD as well as liver-related disease to be the main causes of death in patients with NAFLD. Patients with more advanced fibrosis stage presented increased mortality (HR 3.3, CI 2.27–4.76, *P* < 0.001) [8]. A prospective study including 898 patients that were screened for steatosis by ultrasound found that CVEs, defined as ischemic stroke, myocardial infarction, revascularization procedures, newly diagnosed arterial fibrillation, and cardiovascular death, were associated with NAFLD. The authors also concluded that the presence of NAFLD determined a 2-fold increase in the risk of CVEs. The patients with liver fibrosis presented a higher, 4x increase in the risk for development of CVEs. Kim et al., in a large study comprising 11,154 patients among whom 34% were diagnosed with NAFLD, reported that fibrosis but not NAFLD was associated with increased mortality. CVD represented the main cause of death [79]. A more recent meta-analysis that included 108,711 patients with NAFLD, 44% women and 56% men, showed that CVEs and mortality were twice higher in women than in men (OR 2.12, 95% CI 1.65–2.73, *P* < 0.001) [80].

While simple steatosis alone confers less cardiovascular risk than NASH, the individual overall cardiovascular risk results from the combination of NAFLD stage and cardiometabolic risk factors.

## 4. Cardiovascular Comorbidities in NAFLD

### 4.1. NAFLD and Atherosclerosis

Atherosclerosis is defined by the development of neointimal cholesterotic plaques in large arteries and is directly associated with acute coronary syndrome and stroke.

Several studies have demonstrated that NAFLD is a risk factor for atherosclerosis and, therefore, associated with increased prevalence of ischemic heart disease [81–85]. Atherosclerosis has been extensively documented in patients with NAFLD and subclinical markers of atherosclerosis such as coronary artery calcium (CAC) score [86, 87], as well as carotid intima-media thickness (cIMT) [88–90] or arterial stiffness via brachial-ankle index, have been used to confirm this association. Prospective studies have demonstrated that NAFLD patients are associated with higher CAC scores than those without NAFLD [91, 92], even among patients with normal body mass index (BMI). The annual rate of CAC progression and the cIMT were higher in NAFLD patients independent of obesity, dyslipidemia, or T2DM [86, 93, 94]. Also, increased cIMT was associated with the presence of liver fibrosis assessed by fibrosis-4 (FIB4) and aspartate transaminase to platelet ratio index (APRI) scores [95]. NAFLD is associated with plaques development not only in coronary arteries but also in carotid arteries, iliac arteries, or celiac trunk [96], with predisposition to multiarterial calcifications.

Moreover, NAFLD has also been associated with endothelial dysfunction [97] as well as with unstable coronary plaques [98] explaining the high risk of ischemic events in these patients [3]. Furthermore, patients with ST segment elevation myocardial infarction (STEMI) presented higher short-term mortality and worse long-term prognostic when NAFLD was associated [67].

A meta-analysis including more than 85,000 patients demonstrated that subclinical atherosclerosis was significantly more frequent in those patients diagnosed with NAFLD (OR = 1.60, 95% CI: 1.45–1.78) [89].

NAFLD increases the atherosclerotic risk and makes the patients prone to the development of unstable plaques adding to cardiovascular risk factors as dyslipidemia, obesity, arterial hypertension, and T2DM.

### 4.2. NAFLD and the Cardiac Structure

NAFLD has been associated with structural heart disease. Diastolic dysfunction and heart failure with a preserved ejection fraction and increased myocardial remodeling are common findings in patients with NAFLD [35]. These changes, together with an increased risk of aortic sclerosis [99], can lead to the development of arrhythmias and the increased risk for CVD events [100].

### 4.3. NAFLD and Arrhythmias

NAFLD has been associated with increased risk of atrial fibrillation [101] and prolonged QTc interval [102]. The physiopathological mechanisms that lead to arrhythmias in patients with NAFLD include the increase of the epicardial adiposity which associates a rise in proinflammatory adipocytokines, followed by the development of myocardial fibrosis [35]. Targher et al., in a prospective study including diabetic patients, reported a high risk for atrial fibrillation when NAFLD was associated, with an OR of 4.49 for a 95% CI between 1.6 and 12.9 [101]. Another prospective study comprising patients followed up for 16.3 years reported an independent association between NAFLD and atrial fibrillation, with an adjusted OR of 1.88 for a 95% CI between 1.03 and 3.45 [103]. Ventricular arrhythmias were also associated with NAFLD, in a retrospective study on patients with type 2 diabetes that underwent 24-hour Holter monitoring. After adjusting for confounders, the authors reported an OR of 3.01 for a 95% CI between 1.26 and 7.17 [104].

### 4.4. NAFLD and Hypertension

The relation between NAFLD and hypertension has not yet been fully explained. There are indications that the systemic inflammation associated with NAFLD could promote the activation of the sympathetic nervous system and, thus, induce hypertension [105]. Moreover, insulin resistance would promote hypertension via the augmentation of free fatty acids that lead to perivascular fat deposits situated in the vicinity of vessels and the renal sinus. Furthermore, the high levels of homocysteine found in the setting of NAFLD can, together with gut dysbiosis, induce the increase in oxidative stress and thus promote hypertension [106]. Although several studies demonstrated an association between NAFLD and hypertension [57–59, 107], there was considerable heterogeneity concerning the criteria used for the diagnosis of NAFLD.

While some studies used ultrasonography for the diagnosis of NAFLD [58, 59], others used magnetic resonance imaging (MRI) [108] or surrogate scores such as fatty liver index (FLI) [57]. Lau et al., in a prospective study including 3191 patients from Germany, concluded that the subjects diagnosed with NAFLD presented a higher risk of hypertension than patients without NAFLD, reporting an OR of 3.1 for a 95% CI of 1.7–5.8 [58]. Another larger prospective study from South Korea, including 11,350 male patients, found a higher risk for prehypertension in patients with NAFLD. Interestingly, the risk varied according to NAFLD severity [59]. Huh et al. evaluated the risk for hypertension in a prospective longitudinal study including 1,521 patients without CVD. The authors found that the risk for hypertension was higher in the NAFLD group as diagnosed by FLI and that the risk increased gradually in accordance with the FLI value [57]. More recently, Lorbeer et al., in a study that used MRI in order to measure the hepatic fat fraction, reported an association of the liver fat content with high blood pressure as well as with higher odds of hypertension [107].

### 4.5. CVD Events and Associated Mortality in Patients with NAFLD

Numerous studies and meta-analysis have found CVD events to be associated with NAFLD and CVD-related death has been considered the main cause of mortality in these patients [8, 108]. The risk of death following a CVD event in patients with NAFLD was also analyzed in a recent meta-analysis including 16 studies with a total of 34,043 patients. The pooled results indicated an increased risk for fatal and nonfatal CVD events in patients with NAFLD, with an OR of 1.64, 95% CI 1.26–2.13, but the direct causality between NAFLD and CVD events could not be demonstrated because of the observational design of the studies included [100]. A recent study performed by Paik et al. using mortality data from the National Vital Statistics System multiple-cause mortality data between 2007 and 2016 identified 353,234 patients diagnosed with NAFLD. The authors concluded that CVD was the second most frequent cause of death in these patients, following liver cirrhosis [109].

A recent large retrospective study carried out in Germany and involving 111,492 patients showed an increased risk of myocardial infarction when NAFLD was associated, with a hazard ratio of 2.14 (95% CI 1.59, 2.89) [110]. A comprehensive analysis of 285 patients with biopsy-proven NAFLD monitored for 5.2 years showed that advanced fibrosis was a predictor of CVD events and that the NAFLD fibrosis score was the only noninvasive predictor of CVD [111]. However, there is still some controversy regarding the risk of ischemic events in the setting of NAFLD. A matched cohort study of 18 million Europeans including patients from electronic primary healthcare databases from Italy, Netherlands, Spain, and the United Kingdom did not find an increased risk of myocardial infarction or of stroke in patients with NAFLD [71]. However, the design of the study presented the risk for misclassification of the disease; thus, the results should be interpreted with caution.

## 5. Unmet Needs

Long-term assessment of a larger number of histologically diagnosed patients is needed to understand the causes of mortality in NASH and the direct relation with CVD-related events and mortality. Further studies should focus on the role of NAFLD-associated inflammation as a new cardiovascular risk. Also, future studies should be aimed at unraveling the role of other NAFLD-mediated pathways, such as hepatic inflammation, in the pathogenesis of atherosclerosis.

In addition, it is important to better clarify how NAFLD progression from steatosis to more severe disease influences the metabolic and inflammatory components that may associate this disease with atherosclerosis. Prospective long-term studies with homogeneous diagnostic criteria, considering not only the presence but also the severity of NALFD, are necessary to test if this diagnosis can improve cardiovascular disease risk stratification. The greatest challenge would be to separate it from its aggravating metabolic consequences that characterize the MetS, like atherogenic dyslipidemia.

In the meantime, considering the associated higher cardiovascular risk, weight loss, exercise, and control of concomitant established risk factors for atherosclerosis are mandatory in individuals with NAFLD.

## 6. Conclusions

The causal relationship between CVD and NAFLD remains under investigation, but the strong bidirectional association between CVD and NAFLD warrants clinical intervention in patients with NAFLD to modify metabolic risk factors, including T2DM, dyslipidemia, hypertension, and obesity.

Although current cardiovascular society guidelines have not identified NAFLD as an independent risk factor for CVD despite recent studies suggesting NAFLD's role in incident CVD, vigilant age-appropriate screening and treatment for associated risk factors, including weight loss for obesity, glycemic control for T2DM, and treatment of hypertension and hyperlipidemia, remain prudent strategies that should be supported by clinicians managing patients with NAFLD.

Cardiologists should be aware that patients with CVD may have progressive forms of NAFLD, while hepatologists should be aware that patients with progressive NAFLD have a markedly increased risk of CVD. All physicians should perform correct cardiovascular risk management, in a multidisciplinary setting, all these in the best interest of the patients.

## Figures and Tables

**Table 1:** Current evidence of the association between NAFLD and CVD.

Authors, year	Country	Type of study	Main characteristics	NAFLD diagnostic	Results
Bonnet et al., 2017 [56]	France	Prospective, cohort	2,565 patients, normotensive, followed up for 9 years	GGT, FLI	GGT was associated with incident hypertension (standardized odds ratio: 1.21; 95% confidence interval (1.10–1.34); *P* = 0.0001). FLI predicted incident hypertension in a multivariable model.
Huh et al., 2015 [57]	South Korea	Prospective, cohort	1,521 patients, aged 40–70, followed up for 2.6 years	FLI	10.06% of patients developed hypertension; FLI was associated with baseline blood pressure and was an independent risk factor for hypertension.
Lau et al., 2010 [58]	Germany	Prospective, cohort	3191 patients, aged 20–79, followed up for 11.6 years	US and liver Enzymes	Fatty liver disease was associated with hypertension at baseline and at follow-up, OR 2.8; 95% CI 1.3–6.2 and OR 3.1; 95% CI 1.7–5.8, respectively.
Ryoo et al., 2014 [59]	South Korea	Prospective, cohort	11350 patients, only men, aged 30–59, normotensive, followed up for 5 years	US	58.2% of the participants developed prehypertension, 63.7% of the patients with mild NAFLD, and 70.3% of the ones with severe NAFLD, *P* < 0.001.
Sung et al., 2014, [60]	South Korea	Retrospective, cohort	11448 patients, aged 42.1 ± 6.8, normotensive, followed up for 5 years	US	NAFLD was associated with incident hypertension, after adjustment for multiple confounders [aOR = 1.60 (95% CI 1.30, 1.96; *P* < 0.001)].
Agac et al., 2013 [61]	Turkey	Prospective, cross-sectional	80 patients with acute coronary syndrome	US	NAFLD was present in 81.2% of the patients with acute coronary syndrome; multivariate analysis found NAFLD to be associated with higher SYNTAX score (OR, 13.20; 95% CI, 2.52–69.15).
Agarwal et al., 2011 [62]	India	Prospective, cross-sectional	124 patients with T2DM	US	CAD was diagnosed in 60.5% of the patients with NAFLD and in 45.2% of the ones without NAFLD.
Arslan et al., 2012 [63]	Turkey	Prospective, cross-sectional	151 patients with newly diagnosed CAD, without T2DM	US	NAFLD was diagnosed in 64.9% of the patients. Presence of NAFLD was associated with poor coronary collateral development.
Chan et al., 2014 [64]	Malaysia	Prospective, cross-sectional	399 diabetic patients, mean age 62.8 ± 10.5	US	NAFLD was found in 49.6% of patients but was not associated with IHD.
Chen et al., 2010 [65]	Taiwan, China	Retrospective, cross-sectional	295 patients	US, CT	NAFLD (OR, 2.462; 95% CI, 1.065–5.691) was found to be an independent factor for the risk of coronary artery calcifications.
Chiang et al., 2010 [66]	Taiwan, China	Retrospective, cross-sectional	724 patients	US	NAFLD was found to be an independent predictor for future CVD risk ≥10% (OR: 1.89, *P* = 0.004).
Keskin et al., 2017 [67]	Turkey	Retrospective, cohort	360 patients with STEMI	US	Multivariate analysis found grade 3 NAFLD to be a risk factor for in-hospital mortality (OR 4.2).
Perera et al., 2016 [68]	Sri Lanka	Prospective	120 patients with acute coronary syndrome	US	NAFLD was identified in 46.7% of the participants. NAFLD was associated with a higher predicted in-hospital mortality (adjusted OR 31.3, CI 2.2–439.8, *P* = 0.011) and at 6 months after discharge (adjusted OR 15.59, CI 1.6–130.6, *P* = 0.011).
Wu et al., 2017 [69]	China	Cross-sectional	2345 patients	US	NAFLD was significantly associated with the development of coronary artery calcifications (adjusted OR: 1.348, 95% CI: 1.030–1.765).
Baratta et al., 2020 [6]	Italy	Prospective	898 patients, followed up for 41.4 months	US	Patients with NAFLD presented over 2x increase in risk of CVEs; patients with liver fibrosis had a 4x increase in risk.
Pastori et al., 2020 [70]	Multicenter	Prospective, cohort	1735 patients with nonvalvular atrial fibrillation	FLI	NAFLD was diagnosed in 42.2% of the participants but was not associated with bleeding or with thrombotic risk.
Alexander et al., 2019 [71]	Multicenter (Italy, Netherlands, Spain, United Kingdom)	Matched cohort study	120795 patients with NAFLD or NASH	/	NAFLD was not found to be associated with increased risk for acute myocardial infarction.

NAFLD: nonalcoholic fatty liver disease; FLI: fatty liver index; US: ultrasound; OR: odds ratio; CI: confidence interval; HR: hazard ratio; CAD: coronary artery disease; T2DM: type 2 diabetes mellitus; CAD: coronary artery disease; IHD: ischemic heart disease; CT: computed tomography; CVD: cardiovascular disease; STEMI: ST segment elevation myocardial infarction; CVE: cardiovascular event; NASH: nonalcoholic steatohepatitis.

## Data Availability

The data used to support this study are included within this article.

## Abbreviations

ADMA: Asymmetric dimethyl arginine
ATIII: Antithrombin III
CRP: C-reactive protein
DAMPs: Damaged-associated molecular patterns
Fb: Fibrinogen
IL-6: Interleukin 6
IL-1*β*: Interleukin 1*β*
LDL: Low-density lipoprotein
NLRP3: Pyrin domain-containing protein 3
PAI-1: Plasminogen activator inhibitor-1
PAMPs: Pathogen-associated molecular patterns
PNPLA3: Patatin-like phospholipase domain-containing 3
TM6SF2: Transmembrane 6 superfamily member 2
VLDL: Very low-density lipoprotein
vW: Von Willebrand factor.
